# Draft Genome Sequence of Xanthobacter tagetidis ATCC 700314^T^

**DOI:** 10.1128/MRA.00242-19

**Published:** 2019-07-11

**Authors:** Syeda A. Fatima, Abigail E. Goen, Kyle S. MacLea

**Affiliations:** a Biotechnology Program, University of New Hampshire, Manchester, New Hampshire, USA; b Biology Program, University of New Hampshire, Manchester, New Hampshire, USA; c Department of Life Sciences, University of New Hampshire, Manchester, New Hampshire, USA; University of Delaware

## Abstract

Xanthobacter tagetidis is a thiophene-degrading bacterium associated with root balls of the plant genus *Tagetes*, which includes marigolds. It is a Gram-negative facultatively autotrophic bacterium with pleomorphic morphology exhibiting bent and branching rods. From strain TagT2C^T^ (= ATCC 700314^T^), we report a genome assembly of 4,945,221 bp and a 69.5% G+C content.

## ANNOUNCEMENT

Xanthobacter tagetidis TagT2C^T^ (= ATCC 700314^T^) was isolated from the root balls of the French marigold Tagetes patula and was later found in the root balls and soil compost of other members of the genus *Tagetes*, including the African marigold T. erecta and the South American marigold T. minuta, known in Quechua as wakatay*. X. tagetidis* is a Gram-negative yellow-pigmented facultatively autotrophic bacterium that grows optimally at 28 to 30°C (1). Like other members of the genus *Xanthobacter*, *X. tagetidis* exhibits pleomorphism in its morphology, with its typical rod shape exhibiting substantial irregularity, including bends and branching (1–3). Because the host *Tagetes* species produce copious and diverse thiophene compounds, *X. tagetidis* is metabolically versatile and can grow as a chemolithotrophic autotroph on thiosulfate and other inorganic sulfur compounds, as a heterotroph on thiophene-2-carboxylic acid, acetic acid, and α-ketoglutaric acid, and as a mixotroph on thiosulfate in combination with thiophene-2-carboxylic acid and/or acetic acid (2). This ability has been noted for its potential and demonstrated utility in the biodegradation of sulfur compounds, including anthropogenic pollutants, in the soil (1, 2, 4, 5).

Lyophilized Xanthobacter tagetidis ATCC 700314^T^ was obtained from ATCC (Manassas, VA, USA). Bacteria from a pure culture were grown in tryptic soy broth at 30°C for 48 hours. Genomic DNA was extracted using a DNA minikit (Qiagen, Valencia, CA, USA), and DNA quality and quantity were tested using the DeNovix double-stranded DNA (dsDNA) broad-range assay kit (DeNovix, Wilmington, DE, USA). Purified DNA was fragmented and tagged using the Nextera DNA library prep kit (Illumina, San Diego, CA, USA). The library DNA was then sequenced on an Illumina HiSeq 2500 instrument at the Hubbard Center for Genome Studies at the University of New Hampshire (Durham, NH, USA). The 250-bp reads were trimmed computationally using Trimmomatic (6) in paired-end mode with a window size of 4, quality requirement of 15, and minimum read length of 36. SPAdes version 3.11.0 (7) with default settings was used to assemble the trimmed reads into contigs. After the removal of small contigs of <500 bp, the NCBI Prokaryotic Genome Annotation Pipeline (PGAP) (8) was used to predict genes and annotate them from the 87 contigs in this draft genome of *X. tagetidis*. The genome had a total sequence length of 4,945,221 bp with a 69.5% G+C content, somewhat smaller than its closest sequenced relative, Xanthobacter autotrophicus Py2 (5,308,894 bp and a 67.5% G+C content on the main chromosome). The total number of genes was 4,556, out of which 4,447 were protein-coding genes. It also contained 53 RNA genes (3 rRNAs, 46 tRNAs, and 4 noncoding RNAs [ncRNAs]) and 56 pseudogenes. The largest contig was found to be 613,008 bp long, and the *N*_50_ value was 229,695 bp.

### Data availability.

The Xanthobacter tagetidis ATCC 700314^T^ whole-genome shotgun project has been deposited at DDBJ/ENA/GenBank under the accession number RCTF00000000. The version described in this paper is the first version, RCTF01000000. The raw Illumina data from BioProject PRJNA495830 were submitted to the NCBI Sequence Read Archive (SRA) under the project accession number SRP187584 and the SRA run accession number SRR8670595.
